# GADD45α sensitizes cervical cancer cells to radiotherapy via increasing cytoplasmic APE1 level

**DOI:** 10.1038/s41419-018-0452-x

**Published:** 2018-05-09

**Authors:** Qing Li, Xi Wei, Zhi-Wei Zhou, Shu-Nan Wang, Hua Jin, Kui-Jun Chen, Jia Luo, Kenneth D. Westover, Jian-Min Wang, Dong Wang, Cheng-Xiong Xu, Jin-Lu Shan

**Affiliations:** 1 Cancer Center, Daping Hospital and Research Institute of Surgery, Third Military Medical University, Chongqing, 400042 China; 2 Department of Diagnostic and Therapeutic Ultrasonography, Tianjin Medical University Cancer Institute and Hospital, National Clinical Research Center of Cancer, Key Laboratory of Cancer Prevention and Therapy, Tianjin, China; 3 Department of Radiation Oncology and Biochemistry, University of Texas Southwestern Medical Center, Dallas, TX USA; 4 Department of Radiology, Daping Hospital and Research Institute of Surgery, Third Military Medical University, Chongqing, 400042 China; 5 Department of Thoracic surgery, Daping Hospital and Research Institute of Surgery, Third Military Medical University, Chongqing, 400042 China; 6 State Key Laboratory of Trauma, Burn and Combined Injury, Daping Hospital and Research Institute of Surgery, Third Military Medical University, Chongqing, 400042 China

## Abstract

Radioresistance remains a major clinical challenge in cervical cancer therapy. However, the mechanism for the development of radioresistance in cervical cancer is unclear. Herein, we determined that growth arrest and DNA-damage-inducible protein 45α (GADD45α) is decreased in radioresistant cervical cancer compared to radiosensitive cancer both in vitro and in vivo. In addition, silencing GADD45α prevents cervical cancer cells from undergoing radiation-induced DNA damage, cell cycle arrest, and apoptosis. More importantly, our data show that the overexpression of GADD45α significantly enhances the radiosensitivity of radioresistant cervical cancer cells. These data show that GADD45α decreases the cytoplasmic distribution of APE1, thereby enhancing the radiosensitivity of cervical cancer cells. Furthermore, we show that GADD45α inhibits the production of nitric oxide (NO), a nuclear APE1 export stimulator, by suppressing both endothelial NO synthase (eNOS) and inducible NO synthase (iNOS) in cervical cancer cells. In conclusion, our findings suggest that decreased GADD45α expression significantly contributes to the development of radioresistance and that ectopic expression of GADD45α sensitizes cervical cancer cells to radiotherapy. GADD45α inhibits the NO-regulated cytoplasmic localization of APE1 through inhibiting eNOS and iNOS, thereby enhancing the radiosensitivity of cervical cancer cells.

## Introduction

Cervical cancer is the fourth most common malignant disease^1^ and one of the major causes of cancer-related death among females worldwide^2^. Clinically, radiotherapy is one of the most commonly used treatments for cervical cancer as it significantly reduces the risk of cervical cancer relapse^3^. Over 60% of patients with cervical cancer undergo radiotherapy^4^; however, some cervical cancers develop resistance to radiotherapy, which can significantly compromise clinical outcome. Unfortunately, the mechanism for acquiring and developing radioresistance in cervical cancer remains unclear.

Mechanistically, radiotherapy causes cell cycle arrest and tumor cell death by inducing DNA damage^5^. Thus, aberrant DNA repair is one mechanism whereby cancer cells may become radioresistant. Growth arrest and DNA-damage-inducible protein 45α (GADD45α) is a radiation-inducible gene^6^ that is involved in DNA repair^7, 8^. The effects of GADD45α on cancer cell radiosensitivity have been investigated in several cancer types, but its role in radioresistance remains inconclusive. Lu et al.^9^ and Hur et al.^10^ showed that the inactivation of GADD45α sensitized epithelial cancer cells and hepatoma cells, respectively, to radiation treatment, whereas Zhang et al.^11^ and Asuthkar et al.^12^ reported that the overexpression of GADD45α enhanced the sensitivity of squamous cell carcinoma of the tongue and medulloblastoma cells, respectively, to radiation treatment. Klopp et al.^13^ demonstrated a decrease in GADD45α expression in recurrent cervical squamous cell carcinoma patients. Notably, our group previously found that GADD45α expression was decreased in radioresistant cervical cancer cells^14^. Taken together, these findings implicate GADD45α in the development of radioresistance; however, the function and mechanism whereby GADD45α regulates cervical cancer radiosensitivity remains elusive.

Apurinic/apyrimidinic endonuclease 1 (APE1) is a multifunctional protein involved in DNA repair and gene transcription during the adaptive cellular response to oxidative stress, and APE1 reportedly contributes to the development of therapeutic resistance, tumor aggressiveness, and metastasis^15^. The elevated expression or activity of APE1 is associated with increased resistance to radiation in several cancers, including cervical cancer^16–19^. In addition, inhibition or silencing of APE1 dramatically enhances cancer cell sensitivity to radiotherapy in prostate cancer^20^, colorectal cancer^21^, non-small-cell lung cancer^22^, pancreatic cancer^23^, and hepatocellular carcinoma^24^, suggesting an association between APE1 and radiosensitivity across different cancer types. Recent studies have shown that GADD45α regulates APE1 activity in cancer cells through direct interaction^25, 26^.

Given these findings, we propose that GADD45α regulates APE1 and that reduction of GADD45α contributes to the development of radioresistance in cervical cancer. In this work, we demonstrate that GADD45α levels are inversely correlated with radioresistance in cervical cancer patients. Our data indicate that GADD45α sensitizes tumors to radiotherapy by enhancing radiation-induced cell cycle arrest and apoptosis in cervical cancer cells. In addition, our data illustrate that GADD45α enhances the radiosensitivity of cervical cancer cells through the suppression of cytoplasmic APE1 levels via the inhibition of nitric oxide (NO) production.

## Results

### HeLa-XR is a radioresistant cervical cancer cell line

**Fig. 1:**
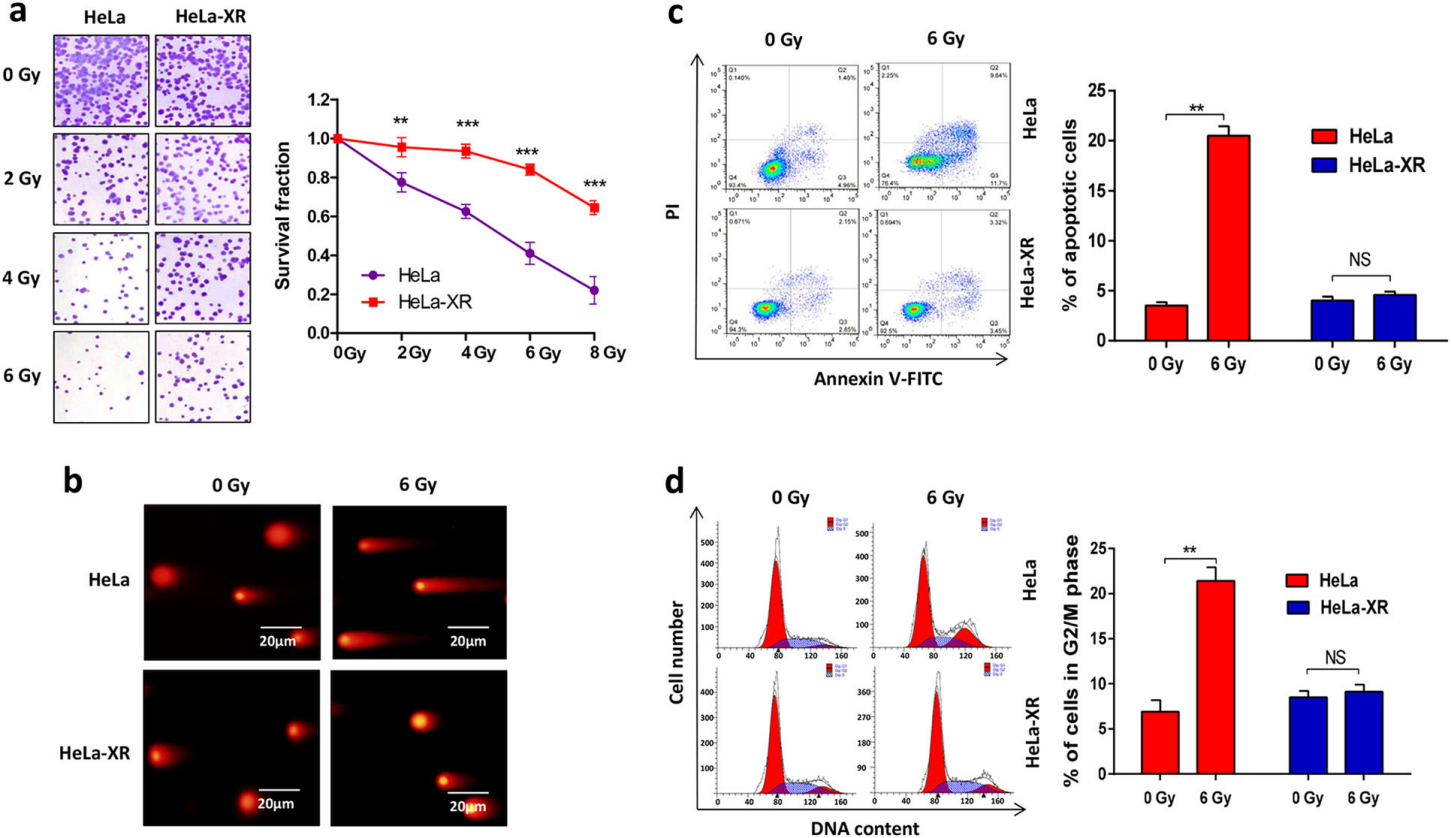
HeLa-XR is a radioresistant cervical cancer cell line. **a** HeLa-XR cells showed higher survival fraction compared to HeLa cells when treated with the same radiation dose. Indicated cells were treated with indicated doses of radiation followed by clonogenic assay. **b** Comet assay showing that HeLa-XR cells were not sensitive to radiation-induced DNA damage compared to HeLa cells. **c** HeLa-XR cells showed reduced apoptotic cell population compared to HeLa cells when treated with 6 Gy radiation. **d** HeLa-XR cells showed resistance to radiation-induced G_2_/M cell cycle arrest compare to HeLa cells. Each experiment was repeated three times. **p < 0.01; ***p < 0.001. NS no significance

First, we confirmed that the X-ray-resistant HeLa cell line (HeLa-XR) is indeed resistant to radiation treatment. As shown in Fig. 1a, a clonogenic assay revealed that HeLa-XR cells exhibited a higher survival fraction compared to parental HeLa cells when treated with the same dose of irradiation (IR). Consistent with the clonogenic assay, a comet assay also illustrated that HeLa-XR cells exhibited reduced DNA damage compared to HeLa cells when treated with the same dose of IR (Fig. 1b). Furthermore, we compared IR treatment-induced cell apoptosis and cell cycle arrest between HeLa-XR and HeLa cells by flow cytometry. As shown in Fig. 1c, d, 6 Gy IR treatment-induced apoptosis and G_2_/M cell cycle arrest in HeLa cells compared controls (0 Gy IR treatment), but HeLa-XR cells did not exhibit these effects. These findings confirm that HeLa-XR is a radioresistant cervical cancer cell line.

### Decreased expression of GADD45α contributes to radioresistance in cervical cancer

**Fig. 2:**
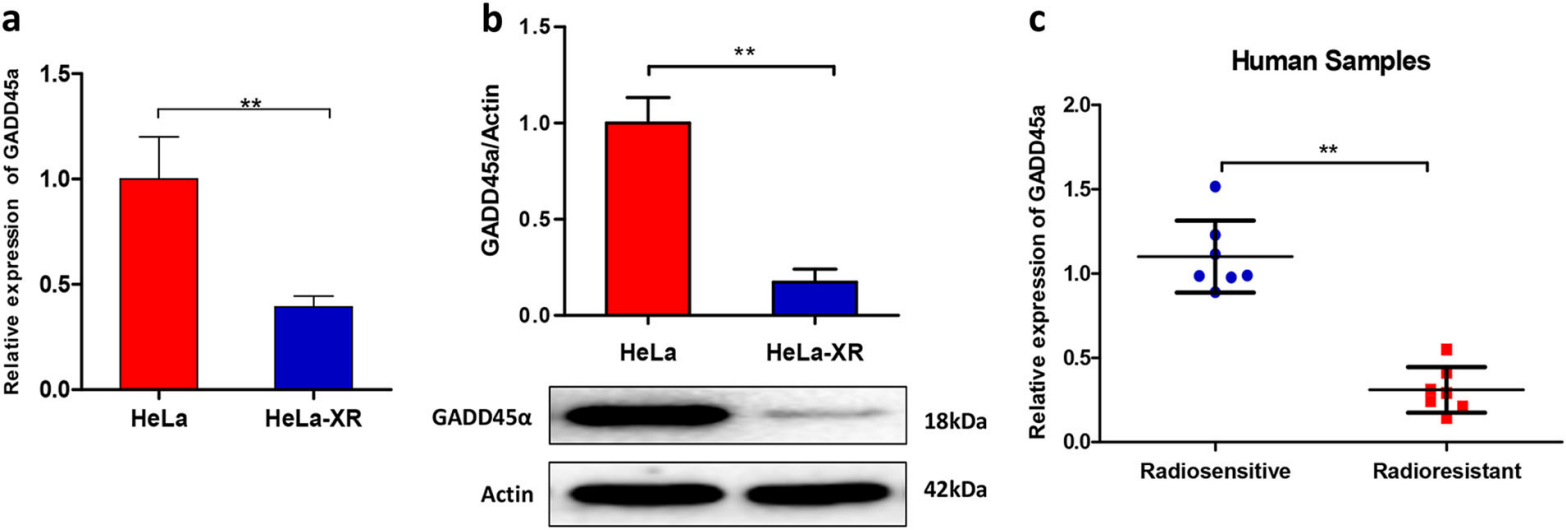
Expression levels of GADD45α are negatively correlated with radioresistance in cervical cancer. **a**, **b** Expression of GADD45α was significantly decreased in radioresistant HeLa-XR cervical cancer cells compared to their parental HeLa cells at both mRNA and protein level. Each experiment was repeated three times. **c** mRNA expression of GADD45α was significantly decreased in specimens from radioresistant cervical cancer patients (*n* = 7) compared to that from radiosensitive cervical cancer patients (*n* = 7). **p < 0.01

**Fig. 3:**
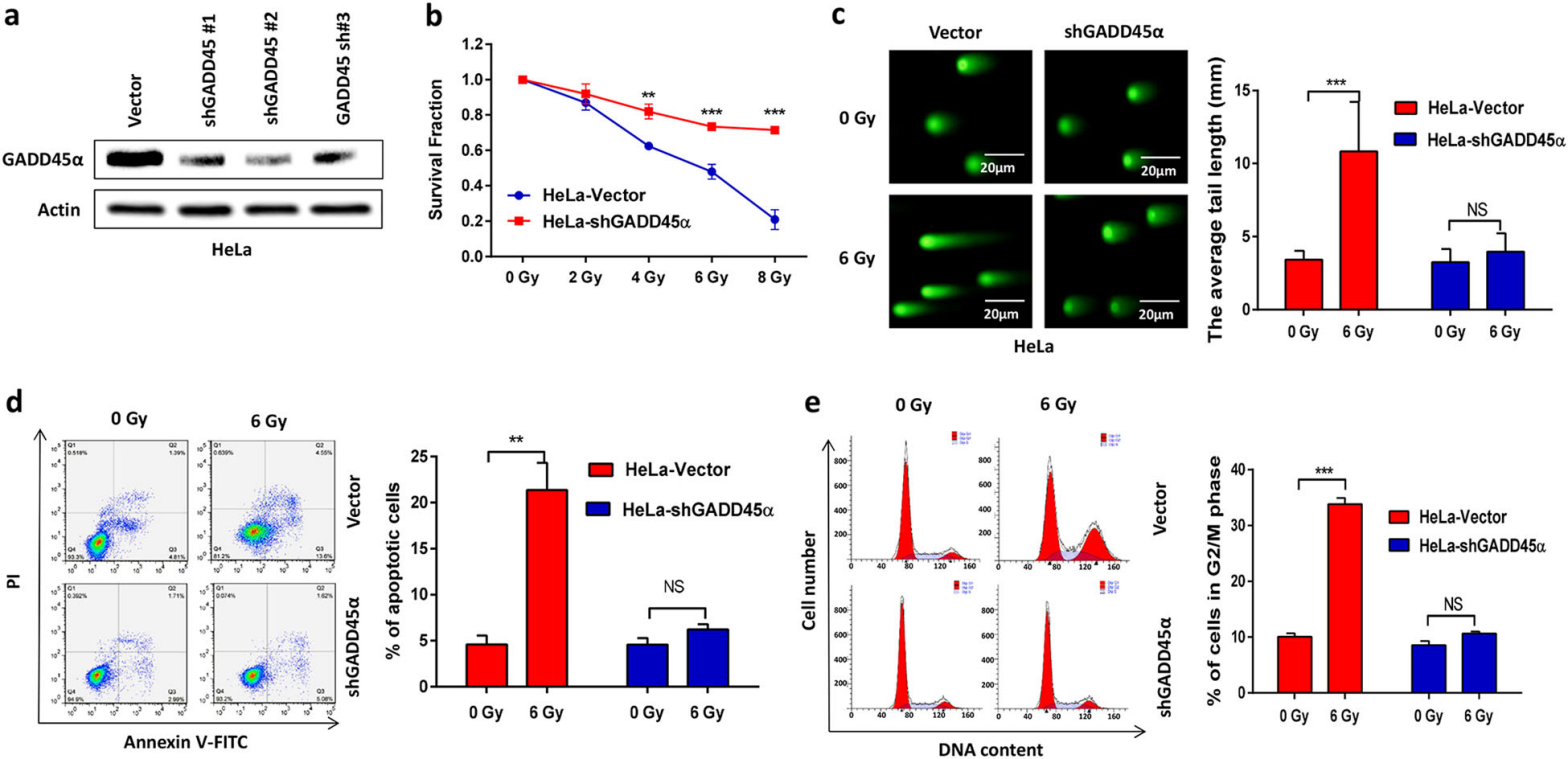
Decreased expression of GADD45α contributes to development of radioresistance in cervical cancer. **a** Transfection of GADD45α shRNA significantly decreased GADD45α expression compared to vector control in HeLa cells. After 72 h of GADD45α shRNA transfection, HeLa cells were subjected to western blot analysis. **b** CCK-8 assay showed that silencing of GADD45α significantly prevented HeLa cells from radiation-induced cell death. **c** Comet assay showed that silencing of GADD45α significantly protected HeLa cells from radiation-induced DNA damage. **d** Apoptosis analysis showed that silencing of GADD45α significantly protected HeLa cells from radiation-induced apoptosis. **e** Cell cycle assay showed that silencing of GADD45α significantly protected HeLa cells from radiation-induced G_2_/M cell cycle arrest. Each experiment was repeated three times. **p < 0.01; ***p < 0.001

Our previous microarray analysis indicated that levels of the DNA damage repair gene GADD45α were significantly reduced in X-ray-resistant HeLa cells^14^. We confirmed these results using Quantitative Real-time Polymerase Chain Reaction, RT-qPCR and western blot analysis in cultured cells and cervical cancer patient specimens. Our data showed that the expression of GADD45α was significantly decreased in radioresistant HeLa-XR cervical cancer cells at both the mRNA and protein level compared to parental HeLa cells (Fig. 2a, b). In agreement with the in vitro experimental results, clinical specimens also presented with significantly reduced GADD45α expression compared to specimens from radiotherapy-sensitive patients (Fig. 2c), suggesting that decreased GADD45α expression correlates with radioresistance in cervical cancer. Next, we investigated the impact of GADD45α on the radiosensitivity of cervical cancer cells. As expected, our data showed that silencing GADD45α (Fig. 3a) dramatically protected HeLa cells from IR-induced cell death (Fig. 3b), DNA damage (Fig. 3c), apoptosis (Fig. 3d), and G_2_/M phase cell cycle arrest (Fig. 3e). Consistent with these results, silencing of GADD45α in the GADD45α-high expressing cervical cancer cell lines CasKi and SiHa (Supplementary Fig. 1) also protected these two cell lines from IR-induced cell death, DNA damage, apoptosis, and cell cycle arrest (Supplementary Figs. 1 and 2). Altogether, these data clearly demonstrate that the suppression of GADD45α significantly contributes to the development of radioresistance in cervical cancer cells.

### Ectopic overexpression of GADD45α overcomes radiosensitivity in radioresistant cervical cancer cells

**Fig. 4:**
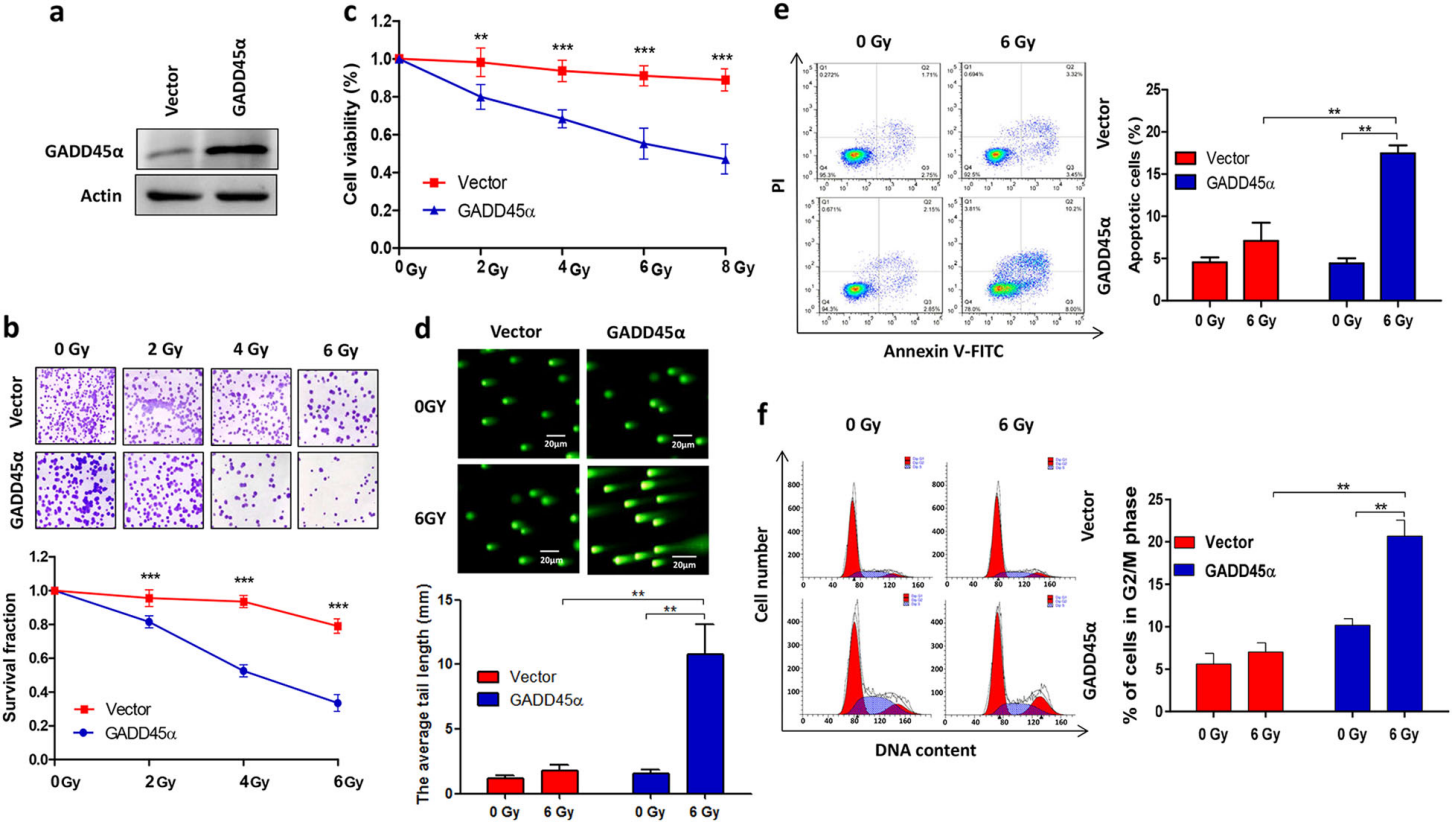
Overexpression of GADD45α enhances sensitivity of radioresistant cervical cancer cells to radiation treatment. **a** GADD45α was significantly increased by transfection of GADD45α expression plasmid in HeLa-XR cells. Western blot analysis was performed 72 h after transfection. **b**, **c** Clonogenic and CCK-8 assays showed that overexpression of GADD45α significantly induced radiation-induced cell death compared to vector controls in HeLa-XR cells. **d** Comet assay showed that overexpression of GADD45α significantly stimulated radiation-induced DNA damage compared to vector controls in HeLa-XR cells. **e** Apoptosis analysis showed that overexpression of GADD45α significantly stimulated radiation-induced apoptosis compared to vector controls in HeLa-XR cells. **f** Cell cycle analysis showed that overexpression of GADD45α significantly stimulated radiation-induced G_2_/M cell cycle arrest compared to vector controls in HeLa-XR cells. Each experiment was repeated three times. The significance between indicated groups was determined using a *t* test. **p < 0.01; ***p < 0.001

**Fig. 5:**
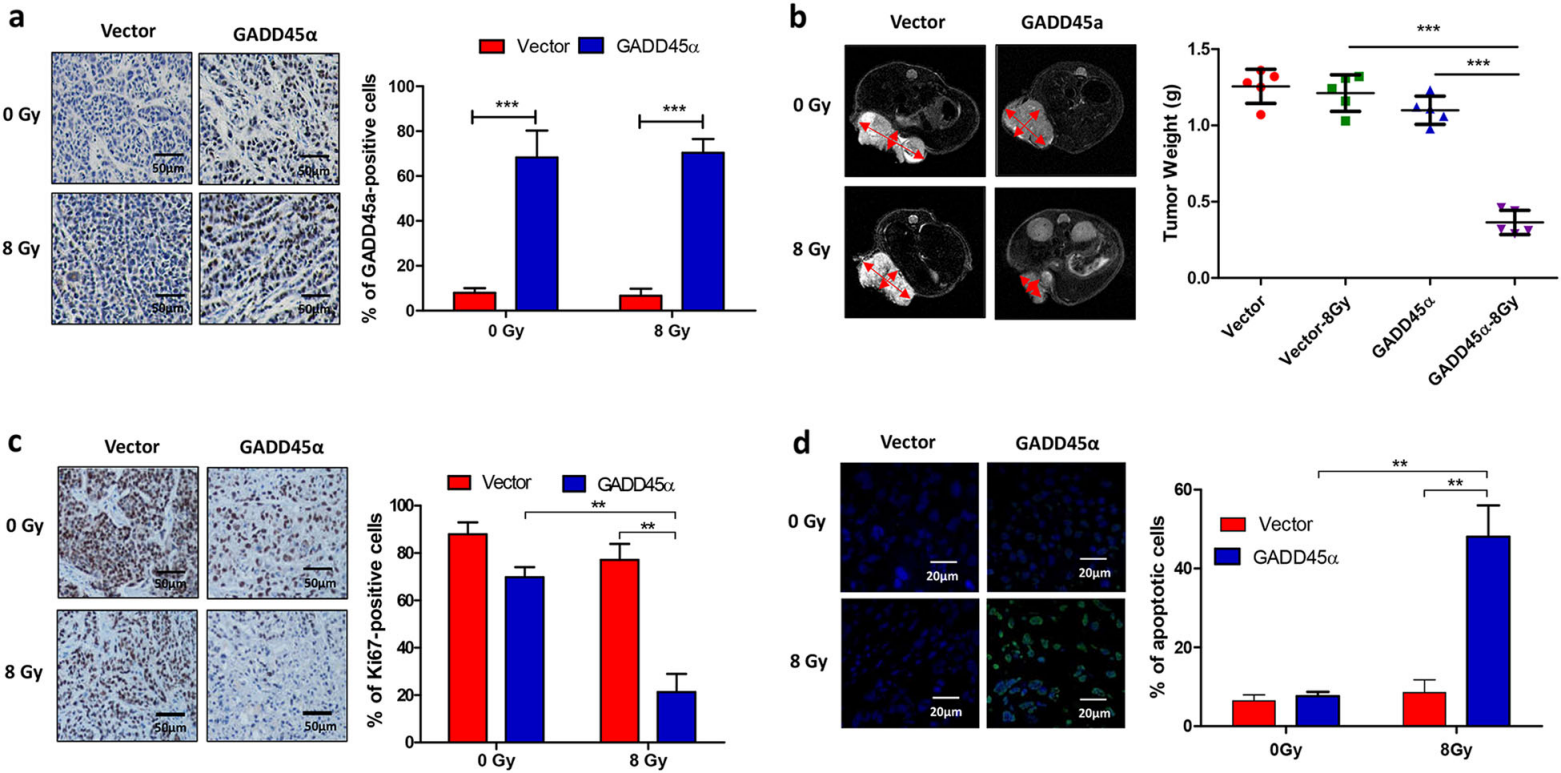
Overexpression of GADD45α overcomes radioresistance in cervical cancer cells in vivo. **a** GADD45α expression levels were examined using immunohistochemistry in a HeLa-XR xenograft model (*n* = 5 per group). **b** Combination of GADD45α overexpression and radiation treatment more significantly inhibited tumor growth compared to control or single treatment (*n* = 5 per group). Tumor volume was measured using MRI. **c** Combination of GADD45α overexpression and radiation treatment more significantly inhibited cell proliferation compared to control or single treatment in vivo. Immunohistochemistry of the cell proliferation marker Ki-67 (*n* = 5 per group). **d** Combination of GADD45α overexpression and radiation treatment more significantly induced cell apoptosis compared to control or single treatment in vivo (*n* = 5 per group). Apoptotic cells were measured using the TUNEL assay. **p < 0.01; ***p < 0.001

Our observation that silencing GADD45α contributes to the development of radioresistance in cervical cancer cells prompted us to investigate whether the overexpression of GADD45α could enhance the sensitivity of radioresistant cervical cancer cells to radiotherapy. To address this, GADD45α was overexpressed in HeLa-XR cells (Fig. 4a), and cells were subsequently treated with 6 Gy IR and subjected to clonogenic, cell viability, apoptosis, cell cycle, and comet assays. As expected, clonogenic (Fig. 4b) and cell viability (Fig. 4c) assays demonstrated that the overexpression of GADD45α significantly enhanced IR-induced cell growth inhibition and cell death compared to vector controls in HeLa-XR cells. Consistent with these results, the comet and flow cytometry analyses showed that the combination of GADD45α overexpression and IR treatment significantly induced DNA damage (Fig. 4d) and apoptosis (Fig. 4e) in HeLa-XR cells compared to IR treatment alone. In addition, cell cycle analysis illustrated that the overexpression of GADD45α with IR exposure considerably increased G_2_/M cell cycle arrest in HeLa-XR cells compared to IR treatment alone (Fig. 4f). Furthermore, we confirmed these in vitro results in vivo using xenograft animal models. As shown in Fig. 5, a single treatment of IR or GADD45α overexpression alone did not significantly inhibit tumor growth, cell proliferation, or induce cell apoptosis, compared to vector controls in the HeLa-XR xenograft model. In contrast, a combination of GADD45α overexpression (Fig. 5a) and IR treatment dramatically suppressed tumor growth (Fig. 5b), with significant inhibition of cell proliferation (Fig. 5c) and induction of cell apoptosis (Fig. 5d) compared to both controls and a single treatment of IR or GADD45α overexpression alone in the HeLa-XR xenograft model. These findings suggest that the overexpression of GADD45α augments radiosensitivity in radioresistant cervical cancer cells via enhancing IR-induced apoptosis and cell cycle inhibition.

### GADD45α reduces cytoplasmic APE1 levels in cervical cancer cells

**Fig. 6:**
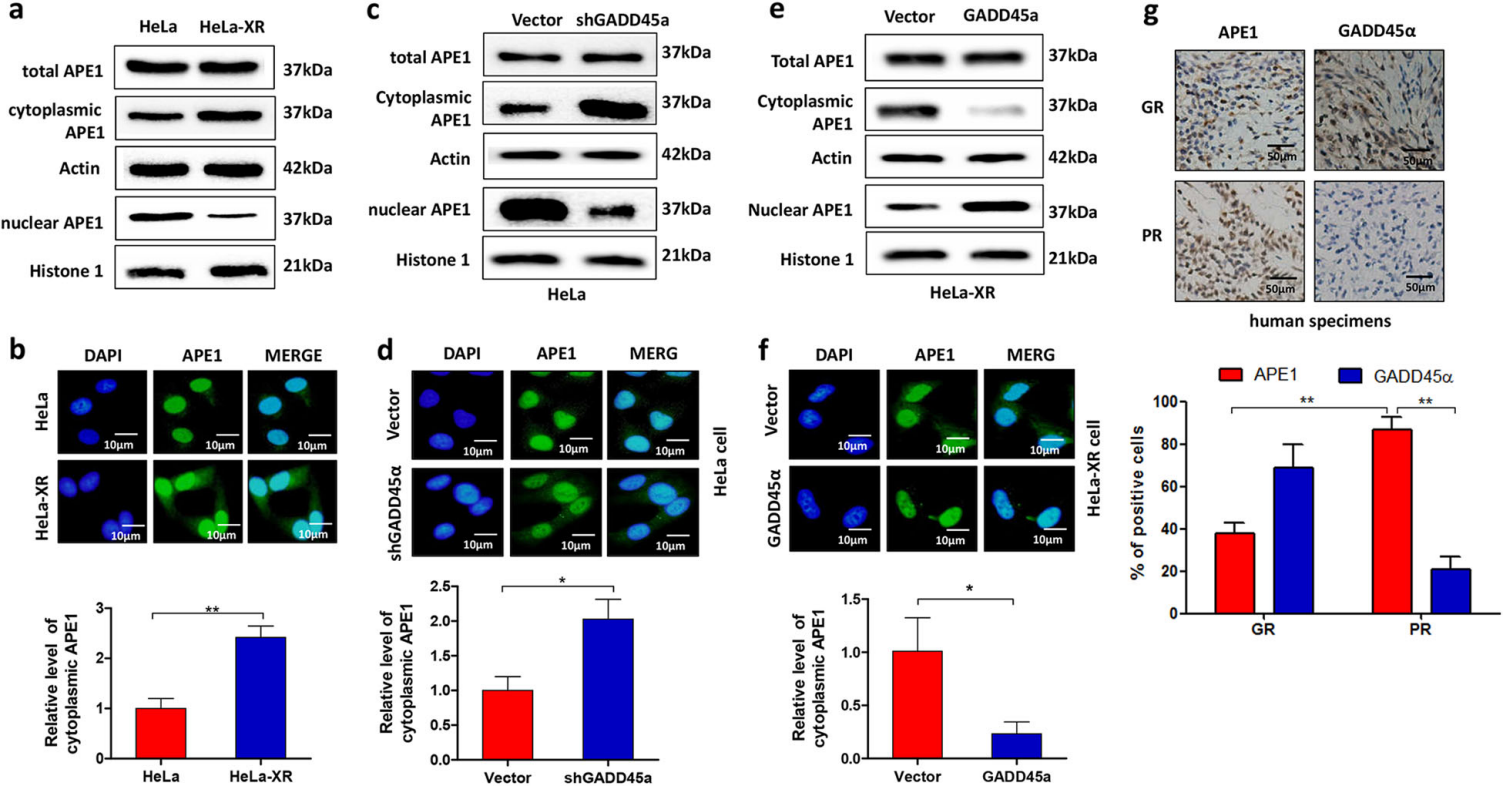
GADD45α negatively regulates cytoplasmic localization of APE1 in cervical cancer cells. **a** Cytoplasmic APE levels were increased, while nuclear APE1 levels were decreased in HeLa-XR cells compared to HeLa cells. Cytoplasmic and nuclear proteins were isolated from indicated cells and the indicated proteins were detected using western blot. **b** APE1 expression and subcellular localization were detected using immunofluorescence in HeLa-XR and HeLa cells. **c** Cytoplasmic APE1 level was increased, while nuclear APE1 level was decreased in HeLa cells in response to silencing of GADD45α. Cytoplasmic and nuclear proteins were isolated from indicated cells after 72 h transfection, and the indicated proteins were detected using western blot. **d** APE1 expression and subcellular localization were detected using immunofluorescence in HeLa cells transfected with shGADD45α plasmid or vector. **e** Cytoplasmic APE1 levels were decreased, while nuclear APE1 levels were increased in HeLa-XR cells in response to GADD45α overexpression. Cytoplasmic and nuclear proteins were isolated from indicated cells after 72 h transfection, and the indicated proteins were detected using Western blot. **f** APE1 expression and subcellular localization were detected using immunofluorescence in HeLa-XR cells that overexpressed GADD45α plasmid or vector. **g** GADD45α expression is negatively correlated with radiation sensitivity and cytoplasmic APE1 expression levels in specimens from cervical cancer patients. Expression of GADD45α and APE1 was measured using immunohistochemistry. Each immunofluorescence experiment was repeated three times. *p < 0.05; **p < 0.01. GR good response, PR poor response

To investigate the mechanism by which GADD45α enhances radiosensitivity, we examined the effects of GADD45α on APE1 expression as APE1 is implicated in cancer radioresistance^27^ and is involved in GADD45α function^25^. Western blot data showed no difference in total APE1 expression levels between HeLa and HeLa-XR cells (Fig. 6a). However, we observed a significant difference in the subcellular distribution of APE1 between HeLa and HeLa-XR cells. Our data showed that radioresistant HeLa-XR cells exhibited higher levels of cytoplasmic APE1 and lower levels of nuclear APE1 than did HeLa cells (Fig. 6a). Similarly, immunofluorescence (IF) showed high levels of cytosolic APE1 in HeLa-XR cells compared to HeLa cells (Fig. 6b). Next, we tested whether GADD45α is involved in the regulation of APE1 subcellular distribution. As expected, western blot and IF data show that silencing of GADD45α did not affect total APE1 expression, but resulted in a significant increase in cytoplasmic APE1 and a significant decrease in nuclear APE1 compared to vector controls in HeLa cells (Fig. 6c, d). In contrast, the overexpression of GADD45α decreased cytoplasmic APE1 levels and increased nuclear APE1 compared to control HeLa-XR cells but did not affect total APE1 expression levels (Fig. 6e, f). Similar to our in vitro data, the clinical data indicated that GADD45α expression is inversely correlated with cytoplasmic APE1 levels and resistance to radiotherapy (Fig. 6g). Collectively, these findings suggest that GADD45α mediates APE1 subcellular distribution in cervical cancer cells.

### GADD45α reduces cytoplasmic APE1 levels through inhibition of NO production in cervical cancer cells

**Fig. 7:**
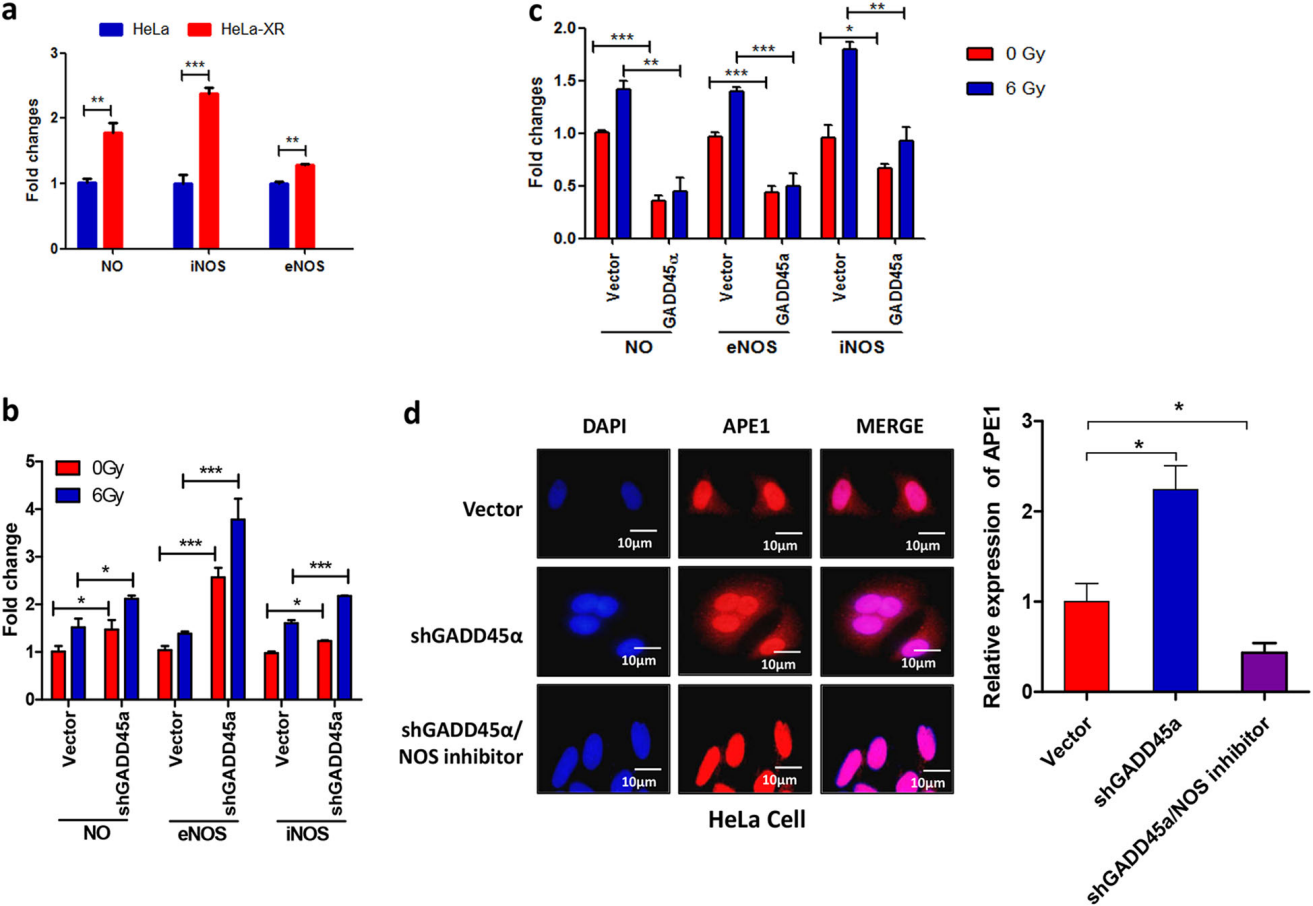
GADD45α inhibits APE1 cytoplasmic localization via suppressing NO production in cervical cancer cells. **a** Levels of NO, eNOS, and iNOS were significantly increased in HeLa-XR cells compared to HeLa cells. Cellular levels of NO, eNOS, and iNOS were measured using an ELISA Kit. **b** Silencing GADD45α significantly increased radiation-induced NO, eNOS, and iNOS in HeLa cells. **c** Overexpression of GADD45α significantly inhibited radiation-induced NO, eNOS, and iNOS in HeLa-XR cells. **d** NOS inhibitor treatment prevented GADD45α depletion-induced cytoplasmic APE1 accumulation in HeLa cells. Subcellular APE1 localization was detected using immunohistofluorescence. HeLa cells that were transfected with GADD45α shRNA or vector were treated with NOS inhibitor. After 12 h inhibitor treatment, immunofluorescence (IF) assays were performed. Each experiment was repeated three times. Red: APE1; blue: DAPI. *p < 0.05; **p < 0.01; ***p < 0.001

To investigate the mechanism of GADD45α-mediated regulation of APE1 subcellular distribution, we examined the effects of GADD45α on the production of NO and the expression of inducible NO synthase (iNOS) and endothelial NO synthase (eNOS). As shown in Fig. 7a, levels of NO, iNOS, and eNOS were significantly increased in HeLa-XR cells compared to parental HeLa cells. Next, we investigated whether GADD45α is involved in the regulation of NOS expression and NO production. Our data demonstrate that silencing GADD45α significantly increases NO production and eNOS and iNOS expression in HeLa cells (Fig. 7b). In addition, silencing GADD45α increases IR treatment-induced production of NO, iNOS, and eNOS more significantly than in vector controls (Fig. 7b). In contrast, the overexpression of GADD45α significantly suppressed NO production and eNOS and iNOS expression in HeLa-XR cells compared to vector controls. In addition, the overexpression of GADD45α significantly inhibited IR-induced NO production and eNOS and iNOS expression in HeLa-XR cells compared to vector controls (Fig. 7c). On the other hand, inhibiting NO production using a NOS inhibitor dramatically suppressed GADD45α-induced cytoplasmic APE1 accumulation (Fig. 7d). Taken together, these findings indicate that GADD45α reduces cytoplasmic APE1 through the inhibition of NO production by reducing both iNOS and eNOS expression in cervical cancer cells.

## Discussion

Radioresistance correlates with poor clinical outcome in cervical cancer patients. Unfortunately, there is no available treatment specific to cervical cancer patients with radioresistance. Herein, we identified that the expression of GADD45α was significantly reduced in radioresistant cervical cancer cells and specimens from radioresistant cervical cancer patients compared to radiosensitive cervical cancer cells and specimens from radiosensitive cervical cancer patients, respectively. In addition, our in vitro and in vivo experimental data show that silencing GADD45α increases the radiosensitivity of radioresistant cervical cancer cells to IR treatment via the inhibition of IR-induced apoptosis and cell cycle arrest. More importantly, our findings demonstrate that ectopic overexpression of GADD45α significantly enhances radiosensitivity in radioresistant cervical cancer cells. These results suggest that the suppression of GADD45α expression is partially responsible for the development of radioresistance and that the ectopic expression of GADD45α is a potential strategy to overcome radioresistance in cervical cancer.

Next, we have examined the mechanism by which GADD45α regulates cervical cancer cell radiosensitivity. A positive correlation between increased total APE1 expression and radioresistance has been previously identified in numerous cancer studies. Recent studies have shown that not only expression level but also dysregulation of the subcellular localization of APE1 is associated with therapeutic resistance and cancer progression. Clinical studies show that cytoplasmic localization of APE1 correlates with increased aggressiveness and poor prognosis in several cancer types, including lung cancer^28^, ovarian cancer^29^, breast cancer^30^, and hepatocellular carcinoma^31^. More importantly, Qing et al.^32^ reported that patients with greater cytoplasmic APE1 expression experienced a shorter survival time compared to those expressing more nuclear APE1 among cervical patients treated with 252Cf radiation. Herein, our data demonstrate that decreased GADD45α results in cervical cancer cell radioresistance, leading to increased cytoplasmic APE1. In contrast, the overexpression of GADD45α enhances radiosensitivity and decreases cytoplasmic APE1 levels in radioresistant cervical cancer cells. Overall, these findings suggest that GADD45α enhances the radiosensitivity of cervical cancer cells by increasing cytoplasmic APE1 localization.

Finally, we identified a novel mechanism by which GADD45α regulates the subcellular localization of APE1 in cervical cancer cells. NO is an important factor in the regulation of APE1 subcellular localization. Qu et al.^33^ reported that increased NO stimulates the nuclear export of APE1, resulting in cytoplasmic APE1 accumulation. Here, our data show that the overexpression of GADD45α dramatically suppresses radiation-induced NO by significantly inhibiting both eNOS and iNOS expression in cervical cancer cells. In addition, the repression of GADD45α-induced cytoplasmic APE1 accumulation was reduced by treatment with an NOS inhibitor, suggesting that GADD45α hinders nuclear APE1 export by inhibiting NO production and increasing cytoplasmic APE1 levels in cervical cancer cells. Moreover, via the regulation of NO production, GADD45α mediates the subcellular localization of APE1 by interacting with APE1 in the nucleus. According to Jung et al.^25^, APE1 nuclear localization was observed in GADD45α wild-type cells, whereas APE1’s distribution became primarily cytoplasmic in GADD45α-deficient cells. In addition, their study showed that GADD45α contributes to the nuclear interaction between APE1 and proliferating cell nuclear antigen (PCNA). Consistent with these results, Kim et al.^26^ also demonstrated that GADD45α plays a critical role in modulating the interaction of PCNA and APE1. Collectively, our findings combined with reports from other groups suggest that GADD45α represses the nuclear export of APE1 to the cytoplasm via inhibiting NO production and stimulating APE1’s interaction with PCNA in cervical cancer cells.

**Fig. 8:**
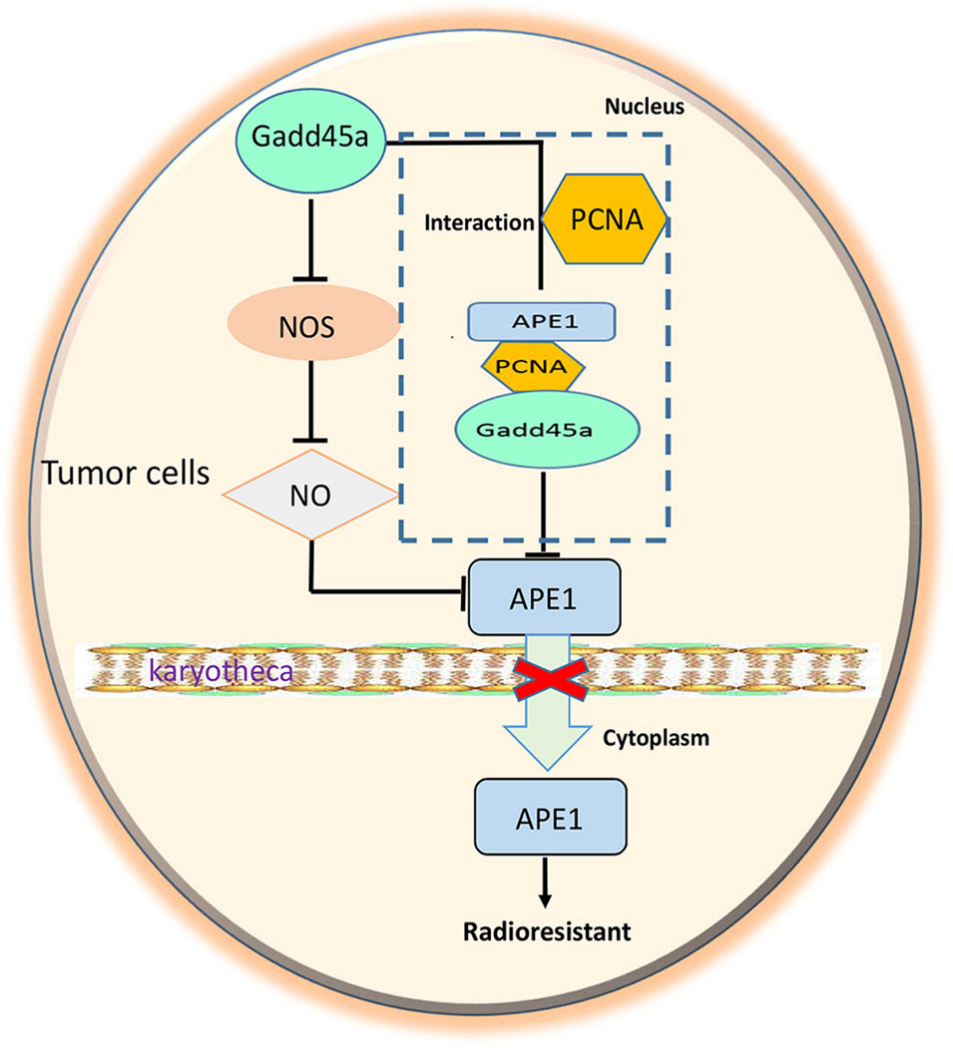
A schematic model of GADD45a effects on the radiosensitivity of cervical cancer

In summary (Fig. 8), this study demonstrates that suppressing GADD45α contributes to development of radioresistance and that overexpressing GADD45α can overcome radioresistance in cervical cancer cells. Furthermore, we demonstrated that GADD45α enhances radiosensitivity by decreasing the cytoplasmic distribution of APE1 in cervical cancer cells. Additionally, we illustrated that GADD45α inhibits APE1 cytoplasmic distribution by inhibiting NO production via the reduction of eNOS and iNOS expression. Further studies are still needed to elucidate the mechanism whereby GADD45α mediates NOS regulation.

## Materials and Methods

### Cell lines and cell culture

The HeLa cell line was obtained from American Type Culture Collection (ATCC, Manassas, VA), and X-ray-resistant HeLa cells (HeLa-XR) were developed using HeLa cells as previously described^14^. HeLa and HeLa-XR cell lines were maintained in Dulbecco’s modified Eagle’s medium with 10% fetal bovine serum (HyClone, Logan, UT) at 37 °C and 5% CO_2_.

### Plasmids and transfection

The GADD45α plasmid and shRNA constructs were obtained from Western Biomedical Technology (Chongqing, China). Cells were transfected with indicated plasmids using Lipofectamine 2000 according to manufacturer’s instructions (Invitrogen, Carlsbad, CA).

### Cell viability and clonogenic assay

To determine cell viability, cells were plated in 96-well plates at a density of 5000 cells per well. Twelve hours after seeding, cells were exposed to indicated doses of IR. After 24 h of IR exposure, cell viability was determined using a CCK-8 kit (Dojindo Laboratories, Kumamoto, Japan) according to the manufacturer’s instructions. For the clonogenic assays, cells were seeded in 60 mm^2^ dishes (1000 cells per plate). Twelve hours after seeding, cells were exposed to indicated doses of IR and were allowed to grow until visible colonies appeared. Colonies were subsequently stained with 0.01% crystal violet (Sigma, St. Louis, MO) and counted under a microscope.

### Apoptosis and cell cycle analysis

Cells were exposed to the indicated doses of IR. After 24 h of IR treatment, cells were subjected to cell cycle and apoptosis analysis. Apoptotic cells were identified by flow cytometry using the Annexin V-FITC kit (Calbiochem, Shanghai, China) according to the manufacturer’s instructions. Briefly, cells were washed twice with cold PBS and resuspended in binding buffer. Next, Annexin V and fluorescein isothiocyanate, FITC were added. Cells were gently mixed followed by incubation for 15 min at room temperature in the dark and then subjected to flow cytometry analysis. For cell cycle analysis, cells were washed twice with cold PBS and fixed in 70% ethanol overnight at −20 °C. Next, the cells were treated with a DNA staining solution, and cell cycle analysis was performed with a Fluorescence activated Cell Sorting, FACS flow cytometer. Apoptotic cells in the tissue were determined using the In Situ Cell Death Detection Kit (Roche, Mannheim, Germany) according to the manufacturer’s instructions.

### Comet analysis

Cells were exposed to the indicated doses of IR. After 4 h of IR, cells were collected and diluted to 4 × 10^4^ cells per ml. Cells were then mixed with 0.5% low melting point agarose and placed into a horizontal electrophoresis tank, and a fresh preparation of cold electrophoresis buffer was added. Next, cells were incubated for 30 min to allow for the formation of DNA chains and then electrophoresed for 25 min (20 V, 300 mA). The glass was washed with a neutral buffer three times (5 min each time) after completion of electrophoresis. The dry glass was stained with 10–15 µl of ethidium bromide (20 µg/ml) and observed by fluorescence microscopy. Comet was analyzed using Komet 5.5 software.

### Real-time quantitative reverse transcription PCR analysis

Total RNA was isolated from cells and fresh tissues with TRIzol reagent (Invitrogen) according to the manufacturer’s protocol. Reverse transcript and real-time PCR assays were performed using a High-Capacity cDNA Reverse Transcription Kit and the QuantiTect SYBR Green PCR kit (Qiagen, Germantown, MD), respectively. The primer sequences for PCR assays were as follows: GADD45α forward, 5′-AGUCGCUACAUGGAUCAAUTT-3′; reverse, 5′-AUUGAUCCAUGUAGCGACUTT-3′; GAPDH forward, 5′-GCAGGGGGGAGCCAAAAGGGT-3′; and reverse, 5′-TGGGTGGCAGTGATGGCATGG-3′.

### Immunohistochemistry, immunofluorescence, and western blot

Immunohistochemistry (IHC), IF, and western blot assays were performed as previously described^34, 35^. Antibodies against APE1, actin, Ki-67, and GADD45α, and secondary antibodies conjugated to horseradish peroxidase were purchased from Abcam (Cambridge, MA).

### Detection of nitric oxide, inducible nitric oxide synthase and endothelial nitric oxide synthase

NO, iNOS, and eNOS were measured in cell lysates using an ELISA Kit according to the manufacturer’s instructions. The Human iNOS ELISA Kit and Human eNOS ELISA Kit were purchased from Wuhan Huamei Biotech Co. (Wuhan, Hubei, China). The NO detection ELISA Kit was obtained from Beyotime Biotech (Shanghai, China).

### Human specimens and animal experiments

Human samples were collected by biopsy from patients before and after radiotherapy. Initial samples were prepared to measure gene expression, and follow-up samples (after radiotherapy) underwent an evaluation to investigate the effectiveness of the radiotherapy. The effectiveness of radiotherapy was evaluated according to methods previously described by Kitahara et al.^36^, and samples were divided into radiosensitive and radioresistant groups based on the results of the evaluation. This study was approved by the Ethics Committee of the Third Military Medical University, and clinical samples were obtained from the Daping Hospital and Research Institute of Surgery at the Third Military Medical University.

Animal experiments were performed using 5-week-old female BALB/c nude mice (Shanghai Laboratory Animal Center, Shanghai, China). Twenty mice were divided into two groups (*n* = 10 per group), and HeLa-XR cells stably overexpressing GADD45α or the empty vector were subcutaneously injected into the left anterior axillae of nude mice (1.5 × 10^7^ cells per mouse). One week following inoculation with the tumor cells, each group was further divided into two groups (*n* = 5 per group), with one group irradiated with 8 Gy every 5 days, three times. Tumor volume was measured using MRI (7.0-T MRI, Bruker Biospec 70/20USR, Germany) every week. On day 35 after inoculation, all mice were killed, and tumors were excised. All animal experiments were approved by the Animal Care Committee of the Third Military Medical University.

### Statistical analyses

All *p* values <0.05 were considered significant. All assays were independently performed at least three times. All values are presented as the mean ± SD. Differences between groups were determined using Student’s *t* test or one-way analysis of variance using the SAS statistical software package version 6.12 (SAS Institute, Cary, NC).

## Electronic supplementary material

suppl figure 1

suppl figure 2

suppl figure 3

Supplementary Figure legends

## References

[1] Ferlay, J. S. I. et al. *GLOBOCAN 2012v1.1, Cancer Incidence and Mortality Worldwide: IARC CancerBase No. 11* (International Agency for Research on Cancer, Lyon, 2015).

[2] Cheng H (2016). Inhibiting CD146 by its monoclonal antibody AA98 improves radiosensitivity of cervical cancer cells. Med Sci. Monit..

[3] Barney BM (2013). Intraoperative electron beam radiotherapy (IOERT) in the management of locally advanced or recurrent cervical cancer. Radiat. Oncol..

[4] Powell ME (2010). Modern radiotherapy and cervical cancer. Int J. Gynecol. Cancer.

[5] Chen Y (2015). IL-6 signaling promotes DNA repair and prevents apoptosis in CD133+ stem-like cells of lung cancer after radiation. Radiat. Oncol..

[6] Papathanasiou MA (1991). Induction by ionizing radiation of the gadd45 gene in cultured human cells: lack of mediation by protein kinase C. Mol. Cell Biol..

[7] Rosemary Siafakas A, Richardson DR (2009). Growth arrest and DNA damage-45 alpha (GADD45alpha). Int. J. Biochem. Cell Biol..

[8] Hollander MC, Fornace AJ (2002). Genomic instability, centrosome amplification, cell cycle checkpoints and Gadd45a. Oncogene.

[9] Lu X (2008). Inactivation of gadd45a sensitizes epithelial cancer cells to ionizing radiation in vivo resulting in prolonged survival. Cancer Res..

[10] Hur JM (2008). Gliotoxin enhances radiotherapy via inhibition of radiation-induced GADD45a, p38, and NFkappaB activation. J. Cell. Biochem..

[11] Zhang XY (2011). Over-expression of Gadd45a enhances radiotherapy efficacy in human Tca8113 cell line. Acta Pharmacol. Sin..

[12] Asuthkar S (2011). Gadd45a sensitizes medulloblastoma cells to irradiation and suppresses MMP-9-mediated EMT. Neuro Oncol..

[13] Klopp AH (2008). Gene expression changes in cervical squamous cell carcinoma after initiation of chemoradiation and correlation with clinical outcome. Int. J. Radiat. Oncol. Biol. Phys..

[14] Qing Y (2010). Microarray analysis of DNA damage repair gene expression profiles in cervical cancer cells radioresistant to 252Cf neutron and X-rays. BMC Cancer.

[15] Wu HH (2013). Cytoplasmic Ape1 expression elevated by p53 aberration may predict survival and relapse in resected non-small cell lung cancer. Ann. Surg. Oncol..

[16] Bobola MS, Blank A, Berger MS, Stevens BA, Silber JR (2001). Apurinic/apyrimidinic endonuclease activity is elevated in human adult gliomas. Clin. Cancer Res..

[17] Sak SC, Harnden P, Johnston CF, Paul AB, Kiltie AE (2005). APE1 and XRCC1 protein expression levels predict cancer-specific survival following radical radiotherapy in bladder cancer. Clin. Cancer Res..

[18] Bobola MS (2011). Apurinic/apyrimidinic endonuclease is inversely associated with response to radiotherapy in pediatric ependymoma. Int. J. Cancer.

[19] Herring CJ (1998). Levels of the DNA repair enzyme human apurinic/apyrimidinic endonuclease (APE1, APEX, Ref-1) are associated with the intrinsic radiosensitivity of cervical cancers. Br. J. Cancer.

[20] Raffoul JJ (2007). Down-regulation of apurinic/apyrimidinic endonuclease 1/redox factor-1 expression by soy isoflavones enhances prostate cancer radiotherapy in vitro and in vivo. Cancer Res..

[21] Xiang DB (2008). Chimeric adenoviral vector Ad5/F35-mediated APE1 siRNA enhances sensitivity of human colorectal cancer cells to radiotherapy in vitro and in vivo. Cancer Gene Ther..

[22] Singh-Gupta V (2011). Soy isoflavones augment radiation effect by inhibiting APE1/Ref-1 DNA repair activity in non-small cell lung cancer. J. Thorac. Oncol..

[23] Chen SM, Xiong GS, Wu SM, Mo JZ (2013). Downregulation of apurinic/apyrimidinic endonuclease 1/redox factor-1 enhances the sensitivity of human pancreatic cancer cells to radiotherapy in vitro. Cancer Biother. Radiopharm..

[24] Cun, Y. P. et al. Silencing of APE1 enhances sensitivity of human hepatocellular carcinoma cells to radiotherapy in vitro and in a xenograft model. *PLoS ONE* **8**(2) e55313 (2013). 10.1371/journal.pone.0055313 PMC3572126 23418439

[25] Jung HJ (2007). Base excision DNA repair defect in Gadd45a-deficient cells. Oncogene.

[26] Kim HL, Kim SU, Seo YR (2013). A novel role for Gadd45alpha in base excision repair: modulation of APE1 activity by the direct interaction of Gadd45alpha with PCNA. Biochem. Biophys. Res. Commun..

[27] Cun Y (2013). Silencing of APE1 enhances sensitivity of human hepatocellular carcinoma cells to radiotherapy in vitro and in a xenograft model. PLoS ONE.

[28] Wu HH (2013). Cytoplasmic Ape1 expression elevated by p53 aberration may predict survival and relapse in resected non-small cell lung cancer. Ann. Surg. Oncol..

[29] Sheng QS (2012). Prognostic significance of APE1 cytoplasmic localization in human epithelial ovarian cancer. Med. Oncol..

[30] Puglisi F (2002). Prognostic role of Ape/Ref-1 subcellular expression in stage I-III breast carcinomas. Oncol. Rep..

[31] Di Maso V (2007). Subcellular localization of APE1/Ref-1 in human hepatocellular carcinoma: possible prognostic significance. Mol. Med..

[32] Qing Y (2009). The expression of APE1 and its correlation with prognostic significance after 252Cf radiotherapy in cervical cancer. Sichuan Da Xue Xue Bao Yi Xue Ban..

[33] Qu J, Liu GH, Huang B, Chen C (2007). Nitric oxide controls nuclear export of APE1/Ref-1 through S-nitrosation of cysteines 93 and 310. Nucleic Acids Res..

[34] Xu CX (2008). Poly(ester amine)-mediated, aerosol-delivered Akt1 small interfering RNA suppresses lung tumorigenesis. Am. J. Resp. Crit. Care.

[35] Xu CX (2008). Chondroitin sulfate extracted from the *Styela clava* tunic suppresses TNF-alpha-induced expression of inflammatory factors, VCAM-1 and iNOS by blocking Akt/NF-kappaB signal in JB6 cells. Cancer Lett..

[36] Kitahara O, Katagiri T, Tsunoda T, Harima Y, Nakamura Y (2002). Classification of sensitivity or resistance of cervical cancers to ionizing radiation according to expression profiles of 62 genes selected by cDNA microarray analysis. Neoplasia.

