# Supplementary figures and images for: GADD45α sensitizes cervical cancer cells to radiotherapy via increasing cytoplasmic APE1 level
Source: Cell Death Dis. 2018 May 9;9(5):524. doi: 10.1038/s41419-018-0452-x (PMC5943293; doi:10.1038/s41419-018-0452-x)

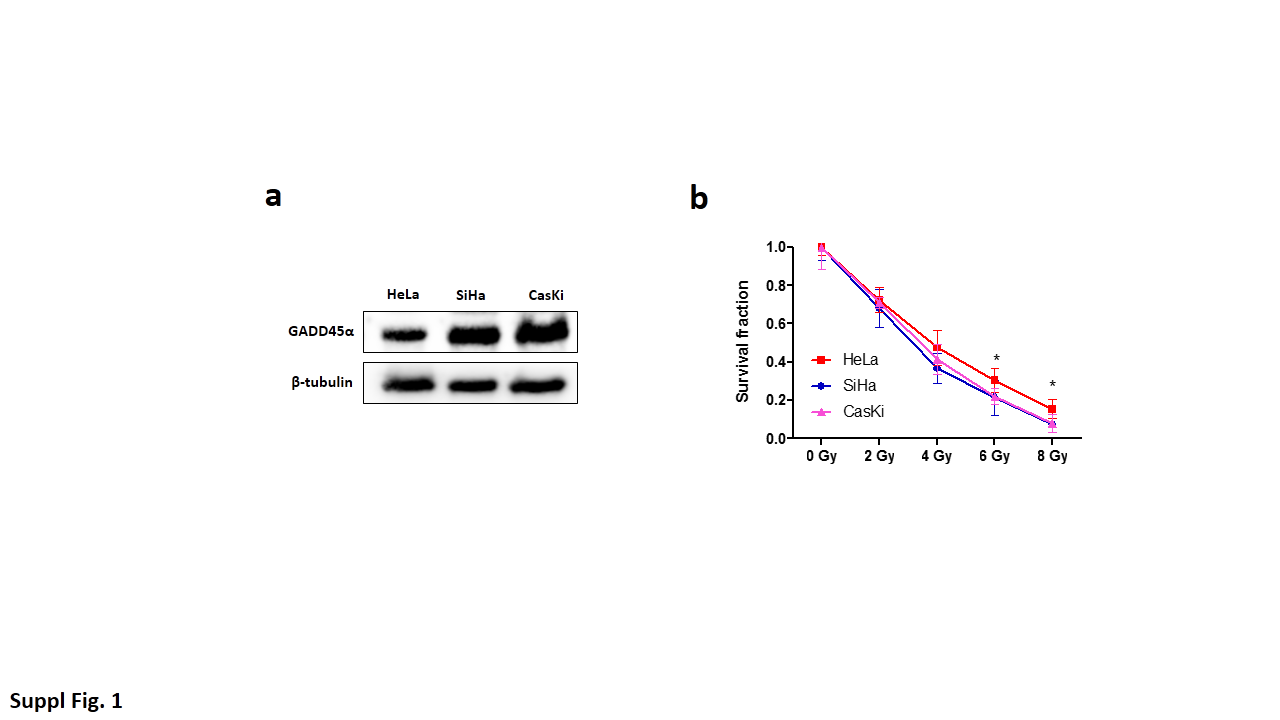

Supplement: Supplementary file 1 — suppl figure 1 [file 41419_2018_452_MOESM1_ESM.tif]

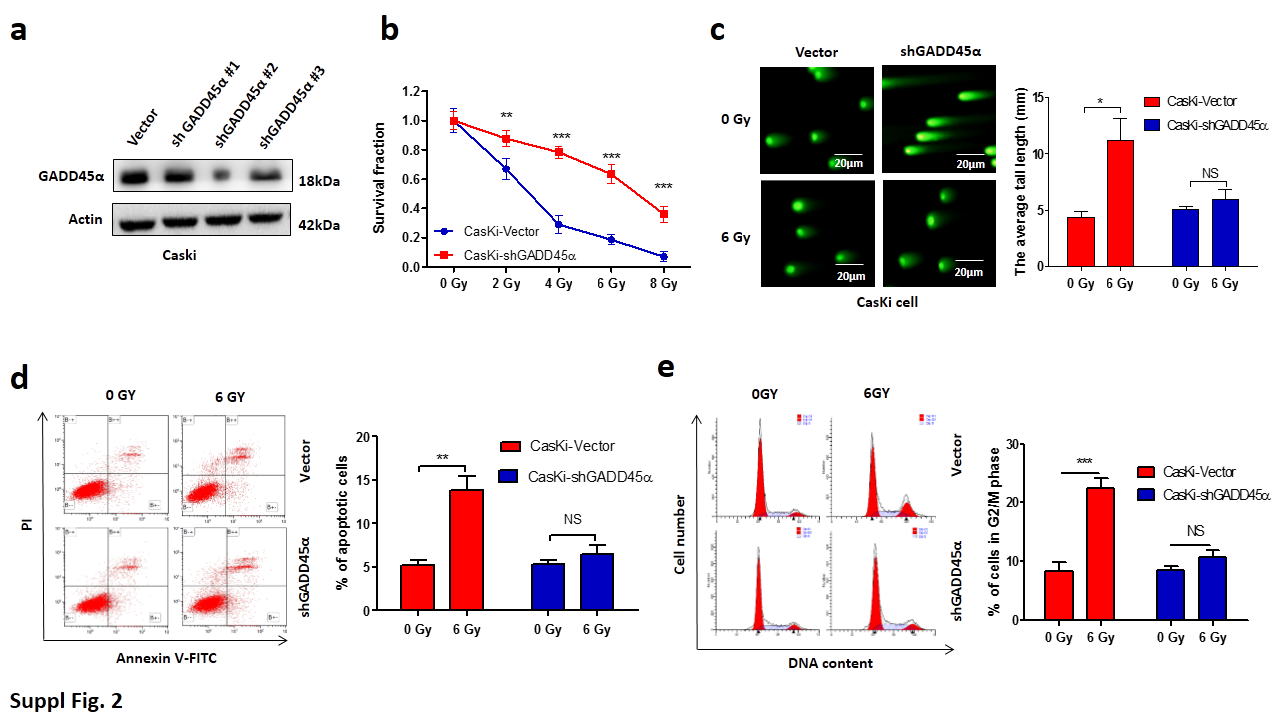

Supplement: Supplementary file 2 — suppl figure 2 [file 41419_2018_452_MOESM2_ESM.tif]

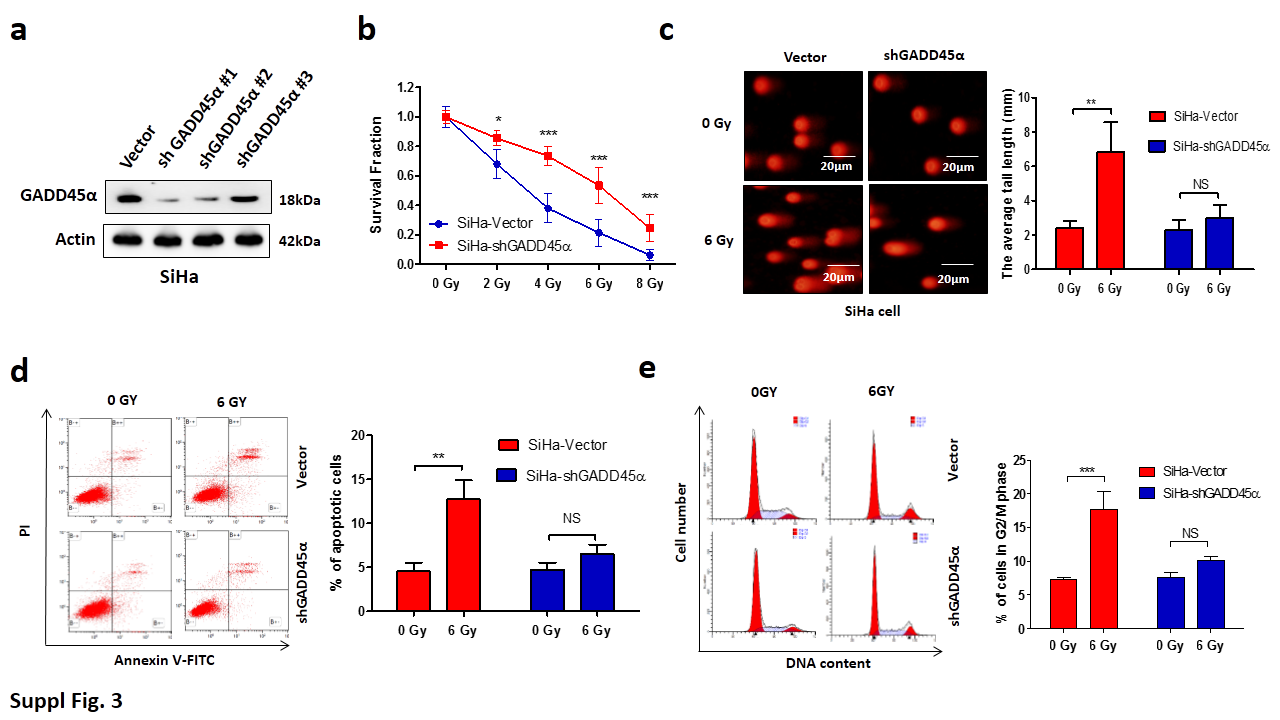

Supplement: Supplementary file 3 — suppl figure 3 [file 41419_2018_452_MOESM3_ESM.tif]
