# Supplementary material for: GADD45α sensitizes cervical cancer cells to radiotherapy via increasing cytoplasmic APE1 level
Source: Cell Death Dis. 2018 May 9;9(5):524. doi: 10.1038/s41419-018-0452-x (PMC5943293; doi:10.1038/s41419-018-0452-x)
Supplement: Supplementary file 4 — Supplementary Figure legends [file 41419_2018_452_MOESM4_ESM.docx]

**Supplementary Figure legends**

**Supplementary Fig 1. Expression level of GADD45α correlated with radiosensitivity in cervical cancer cell lines. a** GADD45α expression was measured using Western blot analysis in the indicated cervical cancer cell lines. **b** Indicated cells were treated with indicated dose of radiation, then, performed clonogenic assay. This experiment was repeated three times. *, *p*<0.01.

**Supplementary Fig 2. Silencing of GADD45α contributes to radioresistance development in CasKi cervical cancer cells. a** Transfection of GADD45α shRNA significantly decreased GADD45α expression compared to vector control in CasKi cells. After 72 hrs of GADD45α shRNA transfection, CasKi cells were subjected to Western blot analysis. **b** GADD45α silenced CasKi cells showed higher survival fraction compared to vector control cells when treated with the same dose radiation. Cells were treated with indicated dose of radiation, then, performed clonogenic assay. **c** Comet assay showed that silencing of GADD45α significantly protected CasKi cells from radiation-induced DNA-damage. **d** Apoptosis analysis showed that silencing of GADD45α significantly protected CasKi cells from radiation-induced apoptosis. **e** Cell cycle assay showed that silencing of GADD45α significantly protected CasKi cells from radiation-induced G_2_/M cell cycle arrest. Each experiment was repeated three times. *, *p*<0.05,**, *p*<0.01; ***, *p*<0.001; NS, no significance.

**Supplementary Fig 3. Silencing of GADD45α contributes to radioresistance development in SiHa cervical cancer cells. a** Transfection of GADD45α shRNA significantly decreased GADD45α expression compared to vector control in SiHa cells. After 72 hrs of GADD45α shRNA transfection, CasKi cells were subjected to Western blot analysis. **b** GADD45α silenced SiHa cells showed higher survival fraction compared to vector control cells when treated with the same dose radiation. Cells were treated with indicated dose of radiation, then, performed clonogenic assay. **c** Comet assay showed that silencing of GADD45α significantly protected SiHa cells from radiation-induced DNA-damage. **d** Apoptosis analysis showed that silencing of GADD45α significantly protected SiHa cells from radiation-induced apoptosis. **e** Cell cycle assay showed that silencing of GADD45α significantly protected SiHa cells from radiation-induced G_2_/M cell cycle arrest. Each experiment was repeated three times. *, *p*<0.05,**, *p*<0.01; ***, *p*<0.001; NS, no significance.
